# Diffuse Traumatic Brain Injury Induced Stimulator of Interferons (STING) Signaling in Microglia Drives Cortical Neuroinflammation, Neuronal Dysfunction, and Impaired Cognition

**DOI:** 10.21203/rs.3.rs-5960640/v1

**Published:** 2025-02-17

**Authors:** Jonathan M. Packer, Samantha G. Giammo, Lynde M. Wangler, Amara C. Davis, Chelsea E. Bray, Jonathan P. Godbout

**Affiliations:** The Ohio State University; The Ohio State University; The Ohio State University; The Ohio State University; The Ohio State University; The Ohio State University

**Keywords:** Microglia, TBI, Inflammation, Cognitive Dysfunction, Stimulator of Interferon Genes, Interferon Type I

## Abstract

Neuropsychiatric complications including depression and cognitive impairment develop, persist, and worsen in the years after traumatic brain injury (TBI), negatively affecting life and lifespan. Inflammatory responses mediated by microglia are associated with the transition from acute to chronic neuroinflammation after TBI. Moreover, type I interferon (IFN-I) signaling is a key mediator of inflammation during this transition. Thus, the purpose of this study was to determine the degree to which a microglia-specific knockout of the stimulator of interferons (STING) influenced TBI-induced neuroinflammation, neuronal dysfunction, and cognitive impairment. Here, microglial inducible STING knockout (CX_3_CR1Cre/ERT2 × STING^fl/fl^) mice were created and validated (mSTING^−/−^). Diffuse brain injury (midline fluid percussion) in male and female mice increased STING expression in microglia, promoted microglial morphological restructuring, and induced robust cortical inflammation and pathology 7 days post injury (dpi). These TBI-associated responses were attenuated in mSTING^−/−^ mice. Increased cortical astrogliosis and rod-shaped microglia induced by TBI were independent of mSTING^−/−^. 7 dpi, TBI induced 237 differentially expressed genes (DEG) in the cortex of functionally wildtype (STING^+/+^) associated with STING, NF-κB, and Interferon Alpha signaling and 85% were attenuated by mSTING^−/−^. Components of neuronal injury including reduced NeuN expression, increased cortical lipofuscin, and increased neurofilament light chain in plasma were increased by TBI and dependent on mSTING. TBI-associated cognitive tasks (novel object recognition/location, NOR/NOL) at 7 dpi were dependent on mSTING. Notably, the TBI-induced cognitive deflcits in NOR/NOL and increased cortical inflammation 7 dpi were unaffected in global interferon-α/β receptor 1 knockout (IFNAR1) mice. In the final study, the RNA profile of neurons after TBI in STING^+/+^ and mSTING^−/−^ mice was assessed 7 dpi by single nucleus RNA-sequencing. There was a TBI-dependent suppression of cortical neuronal homeostasis with reductions in CREB signaling, synaptogenesis, and oxytocin signaling and increases in cilium assembly and PTEN signaling. Overall, mSTING^−/−^ prevented 50% of TBI-induced DEGs in cortical neurons. Collectively, ablation of STING in microglia attenuates TBI-induced IFN-dependent responses, cortical inflammation, neuronal dysfunction, neuronal pathology, and cognitive impairment.

## Introduction

Neuropsychiatric complications including depression and cognitive decline develop and even worsen in the years following traumatic brain injury (TBI). These complications negatively affect quality of life and lifespan. On average, there are 2.4 million brain injuries per year in the United States alone [1]. TBI also increases the risk of dementia and progressive neurodegeneration [2]. Microglia, the innate immune cell of the central nervous system (CNS), are involved in chronic inflammation and progressive neurodegeneration after TBI [3–5]. For example, microglial activation is detected acutely, and evidence of this activation can persist months to years post-TBI in humans [6–9] Microglia and corresponding inflammation after TBI affects brain regions responsible for cognition and executive function, impairing information processing, memory, and executive function [3–5]. Thus, understanding the specific pathways induced in microglia after TBI that promote chronic inflammatory processes is a biologically relevant area of focus.

Myriad reports indicate that TBI-induced chronic neuroinflammation and cognitive dysfunction in rodents are dependent on microglial responses [4, 10–14]. We and others have reported that there is a pronounced shift in microglia RNA profiles from pro-inflammatory and NF-κB mediated genes towards a type 1 interferon (IFN-I) response at a subacute time point after TBI (7 days post injury, dpi) [11, 12, 15]. For instance, single-cell RNA sequencing (scRNA-seq) identified trauma associated microglia 7 dpi with a transcriptional signature that was enriched for IFN-I responses (*Ifitm3, Stat1, Irf7, Ifi27l2a*) [11]. Cortical RNA gene expression also showed the amplification of IFN-I responses 7 dpi [11, 12]. Depletion of microglia prior to TBI using a CSF1R antagonist (PLX5622) attenuated neuroinflammation and ablated the IFN-I response to diffuse TBI. These reductions corresponded with improved cortical dendritic complexity, neuronal physiology, and cognition in novel object location and recognition tasks [11]. Notably, the subacute period after TBI (7 dpi) involved enhanced interferon responses and was worse with age. For instance, diffuse or penetrating TBI in aged mice amplified IFN-I genes (*Ifn-β, Irf7, Ifi204*, and *Isg15*) [16, 17]. In addition, there was amplified IFN-I responses and enhanced gliosis and neuroinflammation in the cortex of aged mice after diffuse TBI [16]. Overall, IFN-I responses are dominant in the brain during the subacute period (7 dpi) after diffuse TBI.

The shift in the inflammatory profile at the subacute period after TBI is pertinent and involves the stimulator of IFN genes (STING). STING is a stress-responsive endoplasmic reticulum protein. In the context of viral infection or injury, tissue damage increases cytosolic double-stranded DNA (dsDNA) and mitochondrial DNA (mtDNA) that are sensed by the cGAS-STING pathway [18, 19]. STING promotes IFN-I responses that enhance transcription factors IRF3, and NF-κB [20, 21] leading to a diverse array of IFN-I and NF-κB-mediated signaling [19]. Type-1 interferons (IFN-α/β) act on the interferon-α/β receptor 1 (IFNAR1) that is expressed by all cell types within the brain. After diffuse TBI, the microglial RNA profile 7 dpi (by snRNA-seq) indicates a STING-dependent production of IFN-I (*cGas, Tbk1, Sting1*). In addition, genes associated with the IFNAR1 (*Ifnar2, Stat1*) and interferon stimulated genes (ISGs) (*Mx1, Mx2, Oasl2*) were also increased in microglia 7 dpi [22]. Consistent with this study, diffuse TBI (lateral fluid percussion injury (FPI)) increased the IFN response in both microglia and astrocytes 7 dpi [15]. In another study, diffuse TBI (midline FPI)-induced STING expression, microglial morphological restructuring, inflammatory, and IFN-related gene expression in the cortex (*Tnf, Cd68, Ccl2, Irf7, Sting*) that was attenuated in global STING^−/−^ mice and by a STING antagonist (chloroquine) [22]. Moreover, TBI-associated cognitive deficits (NOR/NOL) at 7 dpi were STING dependent [22]. Global reductions of STING signaling reduces inflammation, cognitive deficits, and neuronal dysfunction following TBI [22, 23]. Furthermore, a recent study of penetrating TBI using controlled cortical impact (CCI) showed that mSTING^−/−^ reduced the acute inflammatory response, lesion volume, and improved motor recovery 72 hours post injury [24]. Taken together, STING and IFN-I signaling are critical mediators of inflammation, neuronal dysfunction, and cognitive deficits after TBI.

The purpose of this study was to determine the degree to which a microglia-specific knockout of STING influenced neuroinflammation, neuronal dysfunction, and cognitive impairment induced by diffuse TBI. We show novel data that the selective ablation of STING in microglia attenuates TBI-induced IFN-dependent responses, cortical inflammation, neuronal pathology and dysfunction, and cognitive impairment.

## Materials and Methods

### Mice:

To generate inducible CX_3_CR1-STING^−/−^ mice, CX_3_CR1Cre/ERT2 (Jax#:020940) and STING^fl/fl^ (Jax#:035692) mice were purchased from The Jackson Laboratory and bred in-house. Heterozygous offspring were then backcrossed to generate Cre-ERT2 positive (CX_3_CR1Cre/ERT2-STING^fl/fl^) and Cre-negative (STING^fl/fl^) control mice. For genotyping, ear punches biopsies were taken following weaning (21d), and samples were genotyped by TransNetYX (Cordoba, TN). To induce recombination, Cre-ERT2 positive (CX_3_CR1Cre/ERT2-STING^fl/fl^) and Cre-negative control mice (STING^+/+^) were administered 1.5 mg of tamoxifen in 150 ml of corn oil intraperitoneally (i.p.) daily for five days. Mice were allowed 30 days to reconstitute prior to experimental use. The result was a knockout of STING in microglia (CX_3_CR1-STING^−/−^ or mSTING^−/−^) or a functional STING wild type (STING^+/+^). For the global knockout IFNAR1^−/−^ mice, homozygous male and female IFNAR1^−/−^ mice were purchased from the Jackson Laboratory and bred in-house. Male and female mice were used in all experiments unless otherwise noted. These experiments, however, were not powered to make sex comparisons. Mice were group housed under a 12/12 light-dark cycle with *ad libitum* access to food and water. Mice were randomly assigned to groups with mixed treatment and injury groups in each cage. All procedures were performed in accordance with the National Institute of Health Guidelines for the Care and Use of Laboratory Animals, the Public Health Service’s Policy on Human Care and Use of Laboratory Animals, and the Guide for the Care and Use of Laboratory Animals and were approved by The Ohio State University Institutional Laboratory Animal Care and Use Committee.

### Midline Fluid Percussion Injury (mFPI):

Mice were subjected to a midline diffuse TBI using a fluid percussion injury (FPI) apparatus as described previously [12, 22, 25]. Briefly, mice were anesthetized in an isoflurane chamber at 2–3% with a flow rate of 0.8 liters/min. After the surgical site was shaved, mice were secured to the stereotax (Stoelting Co., Cat# 51731) and maintained under anesthesia with a mask attachment (Stoelting Co., Cat# 51609M). The surgical site was prepared with aseptic technique, using alternating applications of iodine and 70% ethanol. Mice received a 3 mm craniectomy between the landmark sutures bregma and λ, and a rigid Luer-loc needle hub was secured over the craniectomy site. Following this procedure, mice were moved to a heated (37°C) recovery cage and monitored until conscious (upright, responsive, and walking). After recovery, mice were briefly re-anesthetized in an isoflurane chamber at 5% (flow rate 0.8 liters/min) for 5 min. The Luer-loc hub was filled with saline, and the hub was attached to the injury device. Once a positive toe-pinch response was elicited (~30 s), a 10 ms pulse of saline (1.2 atm; 670–720 mV) was imposed on the dura. Immediately after the TBI, the Luer-loc hub was removed, dural integrity was confirmed. Next, wound clips (7 mm) were used to close the incision site and the time to self-right was determined (upright and responsive). Next, mice were moved to a heated cage overnight. In these studies, control mice were naïve and uninjured.

### Post-Op Care:

Mice with TBI were monitored for 1 h post-injury and then allowed to recover overnight in a heated recovery cage with accessible food and hydrogel. The next day, mice were returned to their home cages. In these experiments, no analgesics were provided. Mice were weighed and monitored for lethargy (lack of movement) and infection (redness and pus around the incision site) daily throughout the experiments (7 days). Removal criteria included a loss of 20% of baseline bodyweight, sustained lethargy, paralysis, or surgical site infection. In this study, 5 mice were removed based on these criteria.

### Immunohistochemistry and Analysis:

Mice were perfused with phosphate buffered saline (PBS) followed by 4% PFA. Brains were removed, post-fixed, and dehydrated in 30% sucrose. Brains were ash-frozen via isopentane, and then coronal sections (30 μm) were collected, washed, blocked, (0.1% Triton X, 5% BSA, and 5% NDS) and incubated with primary antibodies for rabbit anti-IBA1 (1:1000, Wako, Cat#019–19741, RRID:AB_839504), goat anti-IBA1 (1:500, Wako, #011–27991, RRID:AB_2935833), goat anti-GFAP (1:500, Abcam Cat#ab53554, RRID:AB_880202), rabbit anti-STING (1:200, Proteintech, #19851–1-AP, RRID:AB_10665370), or mouse anti-NeuN (1:500, Abcam, Cat#ab104225, RRID:AB_10711153). Next, sections were washed, incubated with an appropriate fluorochrome-conjugated secondary antibody (donkey anti-rabbit, anti-mouse, or anti-goat; AlexaFluor 488/594/647; Invitrogen) then mounted and cover-slipped with Fluoromount (Beckman Coulter, Inc., Fullerton, CA). Fluorescent labeling was imaged using an EVOS FL Auto 2 imaging system (Thermo Fisher, Waltham, MA). To determine percent area of IBA1^+^, GFAP^+^, STING^+^, or NeuN^+^ labeling, single channel images were converted to 8-bit TIFF format and constant thresholds were used to quantify positively labeled pixels (ImageJ Software). Rod morphology of IBA1^+^ microglia were quantified based on length-to-width ratios as previously described [12]. Values from 4–6 images per mouse were averaged and used to calculate group averages and variance from each group. To determine the number of Lipofuscin^+^ foci, 10 NeuN^+^ cells were selected at random and foci were counted. Lipofuscin (autofluorescence) was detected at 455 nm excitation and 583 nm emission [26]. To determine co-localization of IBA1^+^ and STING^+^ or Lipofuscin^+^ and NeuN^+^ single channel images were converted to 8-bit TIFF format and Just Another Co-localization Plugin (JaCoP) was used to determine the correlation coefficient (ImageJ Software). For colocalization, 4–6 images per mouse were taken using a Nikon Ti2 inverted motorized microscope. Images were taken at 10x magnification as 13 mm z stacks. Denoise.ai was used to reduce background and increase image intensity. Images were analyzed by an investigator blinded to treatment groups.

### NanoString and nCounter Analysis:

NanoString nCounter tissue collection and analysis was performed as previously described [12, 16, 27]. Each experimental group was duplicated in separate experiments, yielding four total experimental groups, with six biological replicates per group. In brief, cortex was collected 7 dpi, flash frozen in liquid nitrogen, and stored −80°C. RNA was isolated using the TRI-Reagent and isopropanol protocol (Sigma-Aldrich). RNA quality and integrity were confirmed using a BioAnalyzer PicoAssay by Chip (Agilent). Gene expression was quantified using the nCounter NanoString neuroinflammation panel targeting 770 genes (https://nanostring.com/). This was performed by the Genomics Core facility at The Ohio State University. Technical normalization was performed to positive and negative controls. Cortical RNA was normalized to the housekeeping gene Csnk2a2. This housekeeping gene was selected based on strong correlation with total counts (*R*^2^>0.8). Differential gene expression analyses were performed using the DESeq2 package in R Studio. Results were generated based on injury, genotype and sex (e.g., TBI-STING^+/+^ vs TBI-mSTING^−/−^). Statistically significant genes had a threshold set to *p*-adj<0.05. Ingenuity Pathway Analysis (IPA, Qiagen) was used to identify canonical pathways associated with the significant genes compared to the respective control (Con-STING^+/+^ or Con-mSTING^−/−^). Results from IPA are represented by *z*-score. Gene names and fold changes were submitted to compare expression patterns in our dataset to IPA’s database. IPA results for canonical pathways (*p*<0.05; composite *z*-score>2) were considered significant. Upstream Regulators were further filtered for activation z-scores (positive or negative) that were associated with either increased or decreased signaling.

### Percoll Enrichment of Brain Myeloid Cells:

CD11b^+^ cells were enriched from whole brain homogenates as described [10, 25, 28]. In brief, brains were manually homogenized using Potter homogenizers, and resulting homogenates were pelleted at 600g for 6 min. Supernatants were removed and cell pellets were resuspended in 70% isotonic Percoll (GE-Healthcare, Catalog #45-001-747). A discontinuous isotonic Percoll density gradient was layered as follows: 50%, 35%, and 0% (PBS). Samples were pelleted for 20 min at 2000 g, and cells were collected from the interphase between the 70% and 50% Percoll layers. These cells were referred to as enriched brain CD11b^+^ cells based on previous studies demonstrating that viable cells isolated by Percoll density gradient yields 90% CD11b^+^ cells [28].

### RNA Extraction and qPCR:

Percoll-enriched myeloid cells were lysed, stored at −80°C, and total RNA was extracted using the Picopure RNA Isolation Kit (ThermoFisher, KIT0204). RNA was normalized by concentration and reverse-transcribed to cDNA. The Applied Biosystems Taqman Gene Expression assay-on-demand protocol and recommended probes for each gene of interest was used for quantitative real-time PCR. Target genes including *Tmem173 (Sting)*: Mm01158117_m1, *H2-Eb1*: Mm00439221_m1, *Irf7*: Mm00516791_g1, *Cd68*: Mm03047343_m1, *Tnf*: Mm00443258_m1, and reference gene *Gapdh*: Mm99999915_g were determined using a QuantStudio 6 (Thermo Fisher) and data were analyzed using the comparative threshold method (ΔΔCt) with data expressed as fold-change from control.

### Novel Object Recognition (NOR) and Location (NOL):

Novel object recognition (NOR) and novel object recognition (NOL) tasks were conducted as previously described [10, 11]. Briefly, these tests involved four 10 min phases each separated by 24 h: habituation (no objects), acclimation (2 objects), recognition (2 objects, with one new object), and location (2 objects, one new location). Discrimination index in the recognition and location trials was determined [(time_novel_-time_familiar_)/time_total_] x100. Videos were analyzed by an investigator blinded to treatment groups.

### Plasma Neuro lament Analysis:

Plasma neurofilament was assessed in duplicate using a Meso Scale Discovery R-PLEX Human Neurofilament L Assay (K1517XR-2) according to the manufacturer’s instructions and as described previously [29]. In brief, mice were euthanized, blood was collected and clarified at 6000 × g for 15 minutes, and plasma was frozen at −80°C until analysis. Neurofilament light chain (NF-L) was analyzed in plasma samples diluted two-fold. The concentration of neurofilament light chain (NF-L) (pg/ml) was determined using the MESO QuickPlex SQ 120 with reference to a standard curve. The standard curve was established using 8 provided calibrator standards (0–50,000 pg/mL). All samples were within the detection range of the standards.

### Nuclei Isolation:

Nuclei were isolated for single nucleus RNA-sequencing as previously described [22]. In brief, each group (*n*=3) was sacrificed simultaneously, then pooled. Each experimental group was duplicated in separate experiments, yielding four total experimental groups, with six biological replicates per group. Cortices were extracted then placed into 2 mL Dounce homogenizers with 1 mL of homogenization buffer. Cortices were homogenized, filtered using a 40 μM strainer and homogenates were clarified. Samples were resuspended in a PBS buffer with RNase Inhibitors (0.05 U/μL of Enzymatics RNAase-Inhibitor and Superase-Inhibitor) and re-pelleted. To remove myelin debris, samples were incubated with Myelin Removal Beads II (Miltenyi Biotec, #130-096-731) for 15 minutes at 4°C. Samples were washed (50% PBS and 50% PBS + 1% BSA) and re-pelleted. Supernatant was removed and samples were resuspended in 1 mL of wash buffer. Two LS columns (Miltenyi Biotec, Cat #130-042-401) were used to filter each of the samples, which were then pelleted and resuspended in 150 μL of wash buffer. Nuclei were counted with AO/PI (Logos Biosystems, #F23001) on a Luna-FL Cell Counter and fixed with a Nuclei Fixation Kit (Parse Biosciences, #SB1003) per the manufacturer’s instructions followed by rapid freezing at −80°C.

### Single-Nuclei Barcoding and Sub-library Generation:

As previously described [22], The Parse Biosciences Whole Transcription Kit was used to barcode and generate eight separate sub-libraries with 12,500 nuclei per sub-library. DNA concentration was measured by Qubit 4 Fluorometer and a Qubit dsDNA HS Assay Kit (Thermo Fisher Scientific, #Q32851). A Bioanalyzer 2100 with a High Sensitivity DNA Assay chip was used to control quality of sub-libraries before samples were sequenced. Based on previous sequencing experiments, RNA was sequenced at a depth of 40,000 reads per nuclei using a NovaSeq S4 at the Advanced Genomics Core at The University of Michigan [22].

### Single-Nuclei Sequencing Data Processing:

Libraries were processed as previously described [22]. In brief, each fastq.gz file was downloaded and aligned to the Genome Reference Consortium Mouse Reference 39 (mm39) using the Parse Biosciences pipeline. Matrices were downloaded and manually filtered in RStudio using Seurat (v4.1.1). Low-quality nuclei and doublets were filtered using Seurat in R. Cell-type identification was done using previously established markers: endothelial cells (*Flt1*), astrocytes (*Slc1a3*), oligodendrocytes (*Mag*), microglia (*Csf1r*), and neurons (*Syt1*). Differential gene expression was performed using the FindMarkers feature of Seurat with non-parametric Wilcoxon rank sum test. Pathway and master regulators analyses were performed with Ingenuity Pathway Analysis (IPA; Qiagen).

### Statistical Analysis:

GraphPad Prism (Version 9; San Diego, CA) was used for analysis of variance (ANOVA) of histological and behavioral data. A Student’s t test was used as appropriate to determine differences between groups. Two-way ANOVA was used as appropriate to determine main effects and interactions between factors. Tukey’s test for multiple comparisonswas used for *post-hoc* analysis when main effects and/or interactions were determined. *p*<0.05 was considered statistically significant. Statistical analysis for snRNA-sequencing using Seurat are described above. Outlier data values were determined using GraphPad Grubbs’ test with an Alpha value of 0.05 selected.

## Results

### TBI-induced STING expression in microglia was ablated by mSTING^−/−^.

**Figure 1:**
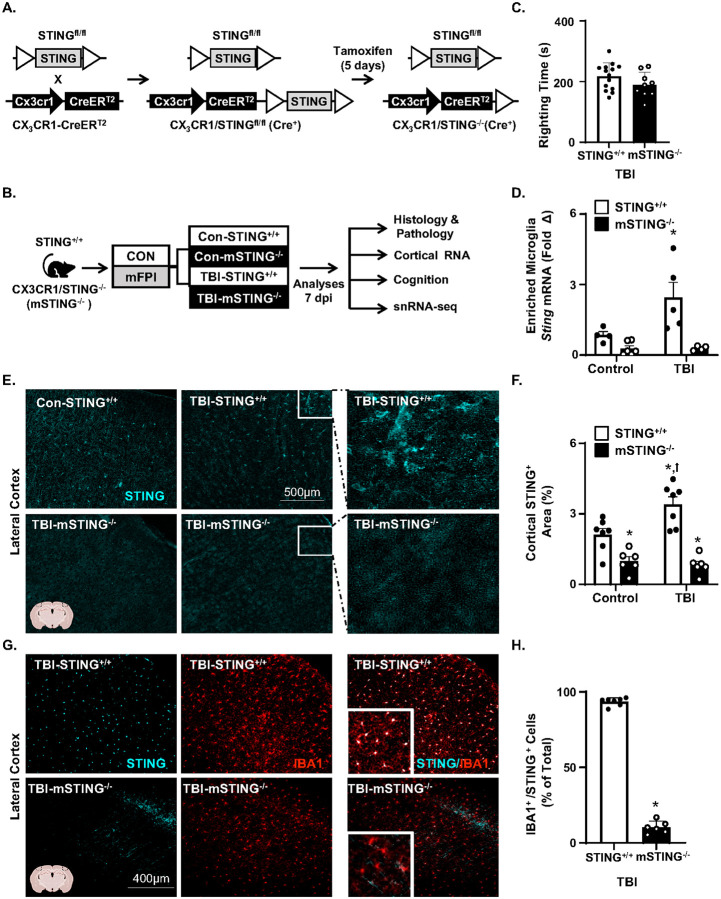
TBI-induced STING expression in microglia was ablated by mSTING^−/−^. A) To generate the inducible and microglia specific knockout of STING, C57BL/6-STING^fl/fl^, mice were crossed with CX_3_CR1-Cre/ERT2 mice. Mice were backcrossed and genotyped to establish CX_3_CR1/STING^fl/fl^ mice (Cre-ERT2^+^) and STING^fl/fl^(Cre-ERT2^neg^) lines. Recombination was induced with tamoxifen (10 mg/ml in corn oil, i.p.) four weeks prior to experiments to generate CX_3_CR1/STING^−/−^ (mSTING^−/−^ mice. B) Male and female functional wild type (STING^+/+^) and mSTING^−/−^ mice were subjected to midline fluid percussion injury or left as uninjured controls. Several parameters were determined 7 dpi including determination of STING (RNA and protein) in microglia. C) Time to self-right for functional wild type (STING^+/+^) and CX_3_CR1/STING^−/−^ (or mSTING^−/−^) mice immediately following TBI (n=9–14). D) *Sting (Tmem117*) RNA determined in percoll-enriched microglia collected from the whole brain 7 dpi (n=4–6). E) Representative images of STING^+^ labeling (10x, 500 mm) in the cortex 7 dpi. Right panels show enlarged images of positive STING labeling in TBI-STING^+/+^ and TBI-mSTING^−/−^ mice. F) Percent-area of STING^+^ labeling in the cortex 7 dpi (n=6–8). From the same experiment, G) Representative images of STING^+^ and IBA1^+^ labeling (n=6–8). G) Right panel shows percent of double labeled IBA1^+^ and STING^+^ cells 7 dpi in the cortex. Bars represent the mean ± SEM, and individual data points are provided. Means with (*) are significantly different from control groups (*p*<0.05) and means with (†) are significantly different from all other groups (*p*<0.05).

We previously reported that a global knockout of STING reduced TBI-associated neuroinflammation and cognitive impairment [22]. The objective here was to understand the cell specificity of STING signaling in microglia after diffuse TBI. First, a transgenic mouse line with an inducible knockout of STING in microglia was created (STING^+/+^ or mSTING^−/−^, Fig.1A). Next, STING^+/+^ and mSTING^−/−^ mice were subjected to control or TBI (mFPI) and several parameters were evaluated 7 dpi (Fig.1B). For instance, time to self-right was assessed immediately after TBI. There were no differences between STING^+/+^ and mSTING^−/−^ mice in self-righting times following TBI (Fig.1C). Next, *Sting* mRNA was determined in percoll-enriched microglia collected from the whole brain 7 dpi (Fig.1D). As expected, there was a main effect of genotype on *Sting* mRNA levels in microglia (*F*_1,15_ = 14.2, *p*<0.005) where mSTING^−/−^ mice had the lowest expression of STING. Moreover, TBI increased STING mRNA in enriched microglia 7 dpi (*F*_1,15_ = 5.0, *p*<0.05), and this was ablated in mSTING^−/−^ mice (Interaction, *F*_1,15_ = 4.8, *p*<0.05). Post-hoc analysis confirmed TBI-STING^+/+^ mice had the highest expression of *Sting* in microglia compared to all other groups (*p*<0.05). These mRNA data help validate the knockout of *Sting* in microglia.

We have also reported that there was increased STING protein and morphological restructuring of microglia and astrocytes 7 dpi after diffuse TBI [22]. Thus, STING protein was assessed in the cortex 7 dpi (Fig.1E–F). Parallel to the mRNA data, TBI increased STING protein expression (*F*_1,22_ = 4.65, *p*<0.05) in the cortex 7 dpi, and this was in uenced by genotype (*F*_1,22_ = 52.10, *p*<0.0001) with less STING protein expression in the mSTING^−/−^ mice compared to STING^+/+^ mice (Fig.1E&F). Post-hoc analyses confirmed that TBI-STING^+/+^ mice had the highest levels of STING in the cortex compared to all other groups including the mSTING^−/−^ mice (*p*<0.001). Parallel to these data, STING expression was determined in IBA1^+^ microglia of the cortex 7 dpi. There was robust expression of STING 7 dpi in IBA1^+^ microglia of wild type (STING^+/+^) mice (Fig.1G&H). Specifically, 94% of IBA1^+^ microglia in the cortex expressed STING after TBI and this expression was reduced to 10% in the mSTING^−/−^ mice (*p*<0.001). Notably, there was non-microglia STING expression detected after TBI (Fig.1F). This increase of STING after TBI, however, was not apparent in cortical astrocytes (GFAP^+^) or neurons (NeuN^+^) (data not shown). These RNA and protein data validate that the mSTING^−/−^ model is working as anticipated. Overall, *Sting* RNA and STING protein were increased in microglia 7 dpi, and both wereablated in microglia from the mSTING^−/−^ mice.

### TBI-induced microglia reactivity 7 dpi was attenuated by mSTING^−/−^.

**Figure 2:**
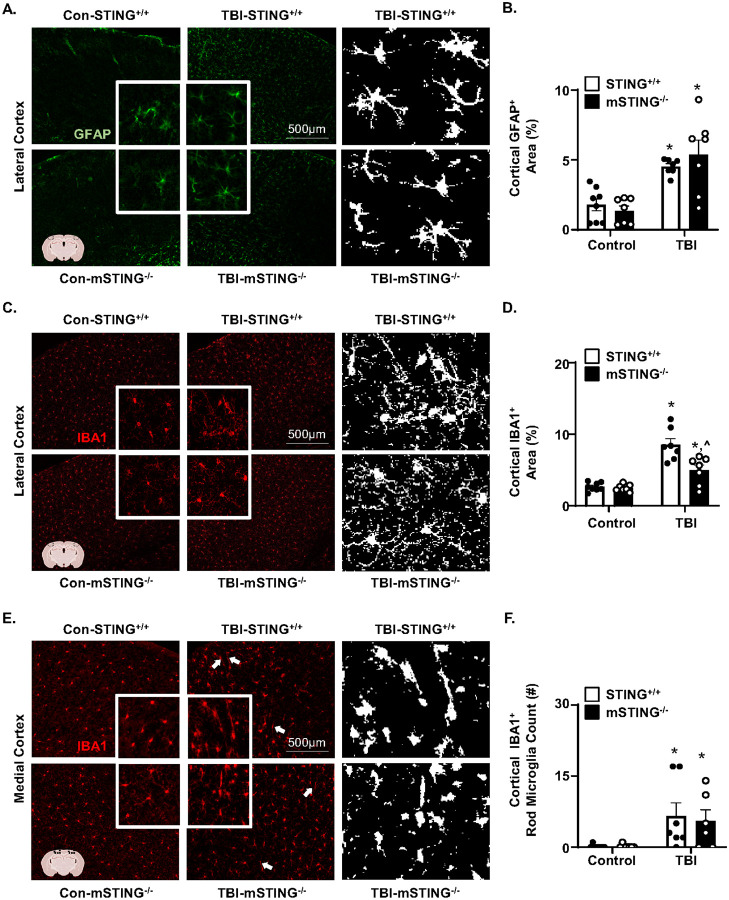
Microglia reactivity 7 dpi was attenuated by mSTING^−/−^. Male and female functional wild type (STING^+/+^) and CX_3_CR1/STING^−/−^ (mSTING^−/−^ mice were subjected to midline fluid percussion injury or left as uninjured controls. A) Representative images of GFAP^+^ labeling (10x) in the cortex 7 dpi. Insets show enlarged labeling and right panel shows pseudo-skeletonized GFAP^+^ labeling (white). B) Percent-area of GFAP^+^ labeling in the cortex 7 dpi (n=6–7). C) Representative images of IBA1^+^ labeling (10x) in the lateral cortex 7 dpi. Insets show enlarged IBA1^+^ labeling and right panel shows pseudo-skeletonized IBA1^+^ labeling (white). D) Percent-area of IBA1^+^ labeling in the cortex 7 dpi (n=6–7). E) Representative images of IBA1^+^ labeling of rod-shaped microglia (10×) in the medial cortex 7 dpi. Insets show enlarged IBA1^+^ labeling and right panel shows pseudo-skeletonized IBA1^+^ labeling (white). F) Number of IBA1^+^ rod microglia per 10× field in the medial cortex 7 dpi (n=6–7). Means with (*) are signi cantly different from control groups (*p*<0.05) and means with (^) are significantly different from TBI-STING^+/+^ mice (*p*<0.05).

Continuing with the influence of mSTING^−/−^ on TBI responses (Fig.1A&B), cortical gliosis and microglial morphological restructuring was determined 7 dpi in male and female STING^+/+^ andmSTING^−/−^ mice. As expected, there was a TBI-dependent increase in the percent area of GFAP^+^ astrocytes (*F*_1,25_ = 33.56, *p*<0.0001, Fig.2A–B). This increase in cortical GFAP^+^ expression 7 dpi was independent of mSTING(Fig.2A&B). For cortical microglia, there was a main effect of TBI on percent area of IBA1^+^ labeling (*F*_1,25_ = 55.46, *p*<0.0001, Fig.2C&D). This increase of cortical IBA1^+^ (percent area) after TBI was influenced by mSTING^−/−^ (Interaction, *F*_1,25_ = 9.30, *p*<0.05). Post-hoc analyses confirmed that TBI-STING^+/+^ mice had the highest IBA1^+^ percent area compared to all groups including the TBI-mSTING^−/−^ mice (*p*<0.05). These increases in IBA1^+^ expression are consistent with “reactive microglia” [30] detected after diffuse TBI [12, 25]. Another aspect of microglial restructuring post-TBI is increased rod-shaped microglia in the cortex [12, 31]. Here, rod-shaped microglia were increased 7 dpi in the medial cortex (*F*_1, 24_ = 11.84, *p*<0.005, Fig.2E&F). The increase in rod-shaped microglia, however, was independent of mSTING. Taken together, the reactive morphological profile of microglia 7 dpi was attenuated by mSTING^−/−^, but astrogliosis and rod-shaped microglia were unaffected.

### TBI-associated cortical in ammation was attenuated by mSTING^−/−^.

**Figure 3:**
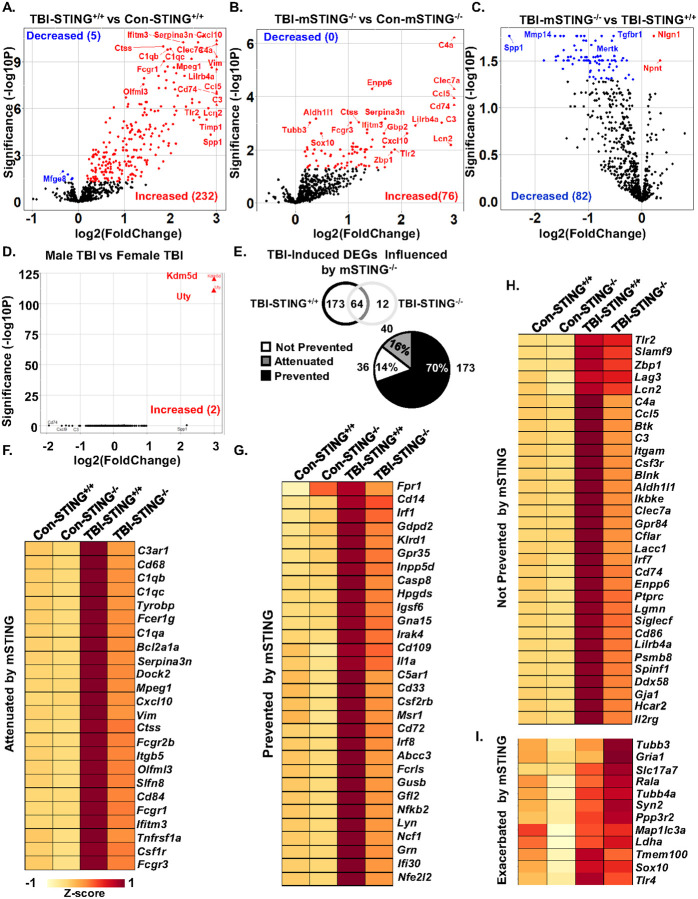
TBI-associated cortical inflammation was attenuated by mSTING^−/−^. A) Male and female functional wild type (STING^+/+^) and CX_3_CR1/STING^−/−^ (mSTING^−/−^ mice were subjected to midline fluid percussion injury or left as uninjured controls. Cortices were collected 7 dpi and mRNA expression was determined using NanoString nCounter analyses (n=6). A) Volcano plot of DEGs in the cortex of TBI-STING^+/+^ versus Con-STING^+/+^ mice. B) Volcano plot of DEGs in the cortex of TBI-mSTING^−/−^ versus Con-mSTING^−/−^mice. C) Volcano plot of DEGs in the cortex of TBI-mSTING^−/−^ versus TBI-STING^+/+^ mice. Red dots represent genes significantly increased with |log2FoldChange| > 0 and *p*-adj<0.05. Blue dots represent genes significantly decreased with |log2FoldChange| > 0 and *p*-ad <0.05. Triangles represent the highest DEGs within any volcano plot. D) Volcano plot of DEGs in the cortex of male TBI–STING^+/+^ versus female TBI-STING^+/+^ mice. E) Percent of genes signi cantly affected by TBI (TBI-STING^+/+^ vs Con-STING^+/+^) that were attenuated, prevented or not prevented in mSTING^−/−^ mice. DEGs that were F) attenuated, G) prevented, H) not prevented, or I) exacerbated are shown in respective heat maps (Fig.3F–I).

Continuing with the assessment of the influence of mSTING^−/−^ on TBI responses (Figs.1&2), cortical inflammation 7 dpi in male and female mice was assessed using NanoString nCounter neuroinflammation panel (770 genes). Genes that were differentially expressed (DEGs) between groups were determined using DESeq2 in R [32]. The first volcano plot (Fig.3A) shows the comparison between TBI-STING^+/+^ and Con-STING^+/+^. There were 232 DEGs increased and 5 DEGs decreased after TBI in this comparison (*p*-adj<0.05). Fig.3B shows the comparison between TBI-mSTING^−/−^ versus Con-mSTING^−/−^. Here, there were 76 DEGs increased and no genes decreased by TBI in mSTING^−/−^ mice (*p*-adj<0.05). Fig.3C shows the comparison between TBI-mSTING^−/−^ mice and TBI-STING^+/+^. In this comparison, there were 2 DEGs increased and 82 DEGs decreased by mSTING^−/−^ (*p*-adj<0.05). As stated above, the TBI response between male and female mice was similar. To highlight this, the TBI comparison between male and female STING^+/+^ mice is shown (Fig.3D). Only two genes (*Kdm5d, Uty*) were differentially expressed after TBI in male and female mice. These DEGs were increased by TBI in males only and are Y-chromosome linked genes [33]. Thus, male and female data were collapsed and analyzed together. Overall, these volcano plots show that TBI induced gene expression in the cortex 7 dpi was robustly influenced by mSTING.

These differences are highlighted in the Venn diagram and pie chart in Fig.3E. The Venn diagram represents DEGs that were uniquely increased in TBI-STING^+/+^ (173 DEGs), shared between the two groups (64) or unique to TBI-mSTING^−/−^ (12). The pie chart shows the percentage of TBI-associated DEGs (249 total) that were attenuated by mSTING^−/−^ (16%, reduced expression), prevented by mSTING^−/−^ (70%, restoration to control levels), or not prevented by mSTING^−/−^ (14%). The majority of the TBI-induced DEGs were attenuated or prevented by mSTING^−/−^. For instance, increased expression of myriad genes that were increased by TBI were either prevented (*Irf1, Ifi30, Ilra Irf8, Nfκb2*) or attenuated (*Itbg5, Itimt3, Olfml3*) by mSTING^−/−^ (Fig.3F&G). Several inflammatory related DEGs were increased by TBI and this increase was either attenuated (*C1qb&c, Cd68 Cxcl10*) or prevented (*Il1a, Cd14, Irak4*) in the TBI-mSTING^−/−^ mice (Fig.3F&G). There were also DEGs induced by TBI (*Tlr2, Tlr4, Irf7, Lcn2*, and *Sox10*) that were not significantly attenuated or prevented by mSTING^−/−^ (Fig.3H&I). Thus, not all inflammatory pathways 7 dpi relied on microglial STING. Overall, a majority of TBI-induced DEGs affected by TBI in the cortex 7 dpi were dependent on STING responses in microglia (86%) while 14% were independent of STING in microglia.

### Canonical pathways associated with inflammation and IFN signaling 7 dpi were attenuated by mSTING^−/−^.

**Figure 4:**
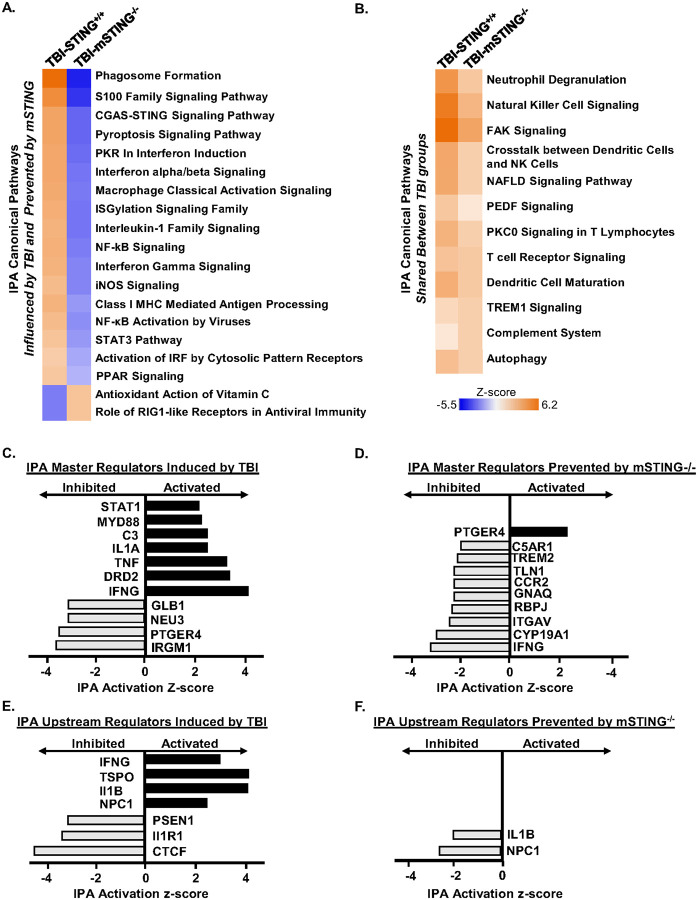
Canonical pathways associated with inflammation and IFN signaling 7 dpi were attenuated by mSTING^−/−^. Continuing with the NanoString experiment outlined in Fig.3, Ingenuity Pathway Analysis (IPA) assessed canonical pathways, master regulators, and upstream regulators in uenced by TBI and mSTING^−/−^ (*z*-score). A) Heat map of IPA canonical pathways induced by TBI. B) Heat map of IPA canonical pathways induced by TBI and prevented by mSTING^−/−^. C) Heat maps of activated or inhibited master regulators by TBI. D) Heat map of activated or inhibited master regulators by TBI and prevented by mSTING^−/−^. E) Heat map of activated or inhibited IPA upstream regulators induced by TBI. F) Heat map of IPA upstream regulators induced by TBI and prevented by mSTING^−/−^. Values were significant with a *p*-adj <0.05.

Continuing with the NanoString analysis, Ingenuity Pathway Analysis (IPA) was used to determine canonical pathways master regulators, and upstream regulators influenced by TBI or mSTING^−/−^. Canonical pathways induced by TBI and prevented by mSTING^−/−^ are shown (Fig.4A). Pathways associated with interferon signaling (cGAS-STING Signaling, Interferon alpha/beta signaling, Interferon Gamma Signaling, Activation of IRF by Cytosolic Pattern Receptors) and neuroinflammation (Phagosome Formation, S100 Family Signaling, Pyroptosis Signaling, Macrophage Classical Activation, NF-κB Signaling, and iNOS Signaling) were all increased in TBI-STING^+/+^ mice (*p*-adj<0.05). Moreover, all these pathways increased by TBI were prevented by mSTING^−/−^ (*p*-adj<0.05). Canonical pathways induced by TBI and unaffected mSTING^−/−^ are also shown (Fig.4B). These mSTING independent pathways included Neutrophil Degranulation, Complement System, Autophagy, and TREM-1 signaling.

Top master regulators induced by TBI and prevented by mSTING^−/−^ are shown (Fig.4C–D). Master regulators associated with interferon signaling (STAT1, MYD88, IFNG) were increased by TBI while regulators associated with neuronal health (GLB1, NEU3, PTGER4, IRGM1) were decreased. Master regulators prevented by mSTING^−/−^ include pathways associated with IFN-I (IFNG), neuroinflammation (C5AR1, TREM2, CCR2, IL1B) and neuronal health (LCP1, PTGER4). Next, upstream regulators induced by TBI and prevented by mSTING^−/−^ are shown (Fig.4E–F). Upstream regulators induced by TBI and prevented by mSTING^−/−^ included pathways associated with phagocytosis (NPC1) and neuroinflammation (IL1B). Taken together, TBI induces myriad inflammatory and interferon mediated genes that were dependent on STING in microglia.

### TBI-induced cognitive deficits 7 dpi were IFNAR1 independent.

**Figure 5:**
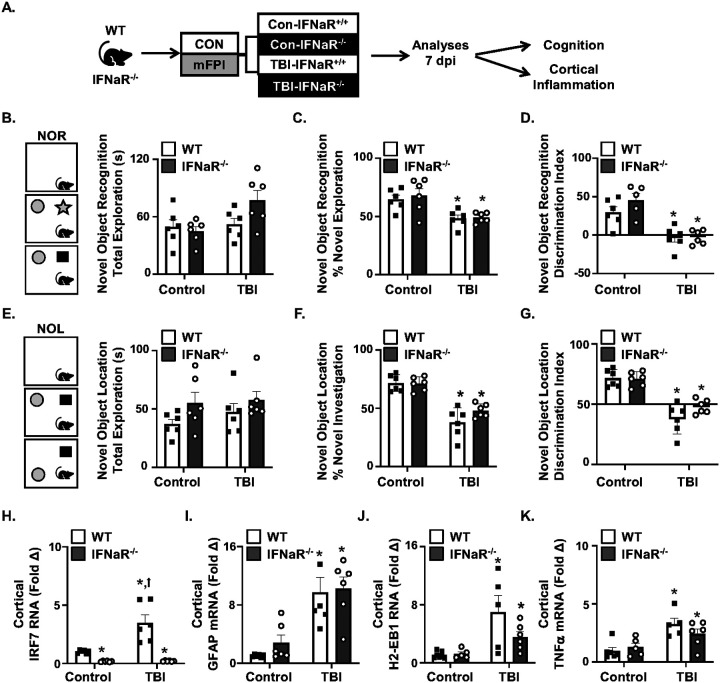
TBI-induced cognitive deficits 7 dpi were IFNAR1 independent. A) Wildtype (IFNAR1^+/+^) and global IFNAR1 knockout (IFNAR1^−/−^) male mice were subjected to midline fluid percussion injury (TBI) or left as uninjured controls. Cognition (novel object recognition (NOR) and location (NOL)) and cortical inflammation were assessed 7 dpi (n=5–6). B) Total exploration time (seconds) of the objects in NOR. C) Percent time exploring the novel object and D) Discrimination index of time exploring the novel object. E) Total exploration time (seconds) of the objects in NOL. F) Percent time exploring the object in the novel location. G) Discrimination index of time exploring the object in the novel location. From the same mice, mRNA levels of H) *Irf7*, I) *Gfap*, J) *H2-Eb1*, and K) *Tnf* were determined from the cortex (n=5–6). Bars represent the mean ± SEM, and individual data points are provided. Means with (*) are significantly different from control groups (*p*<0.05) and means with (†) are significantly different from all other groups (*p*<0.05).

Our previous [22] and current data show that both global and microglia-selective STING knockouts reduce inflammation and support cognitive recovery after TBI. Another aspect of the cGAS-STING pathway is the interferon-α/β receptor 1 (IFNAR1), which is the primary receptor for type I interferons, alpha and beta [34–36]. These pathways were apparent in the NanoString analyses at 7 dpi in the cortex (Fig.4). Thus, we next examined components of cognition and inflammation in male global IFNAR1^−/−^ mice. These mice were subjected to control or TBI (mFPI) and cortical and hippocampal-mediated cognition was assessed 7 dpi using NOR/NOL (Fig.5A–F). There were no differences in total time exploring the objects between groups (Fig.5B). Fig.5C&D show there were TBI-induced deficits in NOR 6 dpi with reduced time exploring the novel object and impairments in the discrimination index (TBI, *F*_1,20_ = 20.83, *p*<0.001), but these effects were independent of IFNAR1. These effects were mirrored in the NOL task 7 dpi (Fig.5E–G). Time spent with the object in the novel location and discrimination index were reduced by TBI (F_1,20_ = 70.15, *p*<0.0001, Fig.5E–G), but again these effects were independent of IFNAR1.

**Figure 6:**
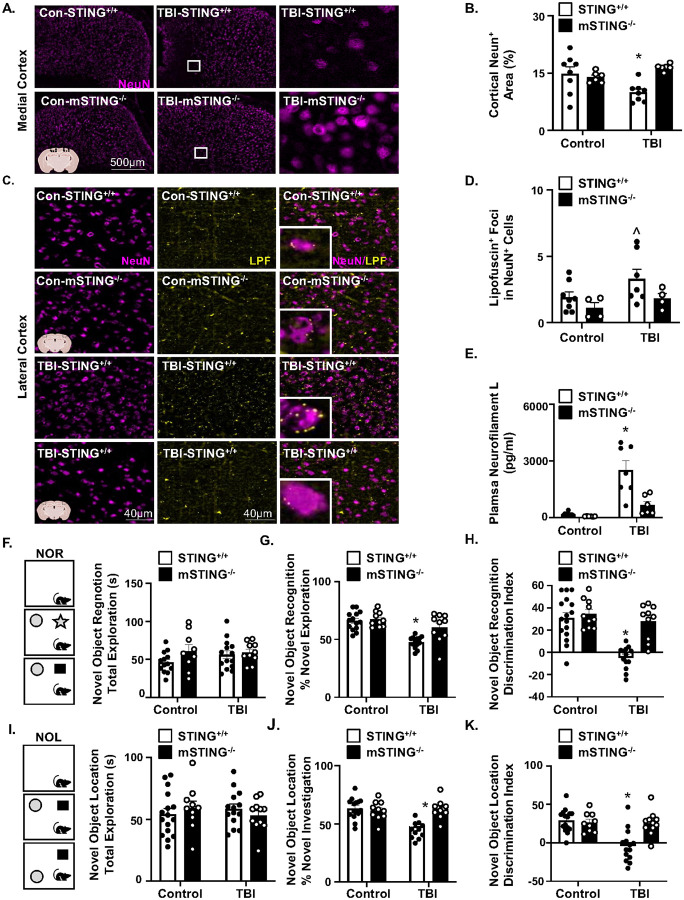
TBI-induced neuronal injury and cognitive deficits 7 dpi were mSTING dependent. A) Male and female functional wild type (STING^+/+^) and CX_3_CR1/STING^−/−^ (mSTING^−/−^ mice were subjected to midline fluid percussion injury or left as uninjured controls. Several parameters of neuronal injury were assessed in the cortex and plasma 7 dpi. A) Representative images of NeuN^+^ labeling (10x) in the cortex 7 dpi. White box represents location where inset was selected. Right panel shows enlarged labeling from inset. B) Percent-area of NeuN^+^ labeling per 10x field in the medial cortex 7 dpi (n=6–8). C) Representative images of lipofuscin autofluorescence and NeuN^+^ labeling in the lateral cortex 7 dpi. Insets show enlarged images of lipofuscin auto-fluorescence in NeuN^+^ cells. D) Quantification of lipofuscin foci in NeuN^+^ neurons in the lateral cortex 7 dpi. E) Neurofilament light chain protein (NF-L) was determined in the plasma 7 dpi (n=6–8). In a separate study, cognition was determined using the novel object recognition (NOR) and location (NOL) tests (n=12–16). F) Total exploration time (seconds) of the objects in NOR. G) Percent time exploring the novel object. H) Discrimination index of time exploring the novel object. I) Total exploration time (seconds) of the objects in NOL. J) Percent time exploring the object in the novel location. F) Discrimination index of time exploring the object in the novel location. Bars represent the mean ± SEM and individual data points are provided. Means with (*) are significantly different from control groups (*p*<0.05). Means with (^) tend to be different from control groups (*p*=0.06–0.1).

After completion of the NOR/NOL cognitive assessment in these mice, cortices were extracted 7 dpi for RNA analyses. TBI increased interferon receptor-related *Irf7* expression, and this increase was dependent on IFNAR1(Interaction, *F*_1,20_, = *p*<0.0017, Fig.5H). Post-hoc analysis confirmed that *Irf7* expression was highest in the TBI-WT group compared to all other groups including the TBI-IFNAR1^−/−^ group (*p*<0.0001, Fig.5H). These *Irf7* data are consistent with the global knockout of IFNAR1. Moreover, several genes associated with inflammation (*Tnf, Gfap, H2-eb1*) were increased in the cortex 7 dpi (TBI, *F*_1,18_ = 7.92, *p*<0.05, for each, Fig.6I–K). The increases in these mRNA levels in the cortex 7 dpi, however, were independent of IFNAR1. Collectively, TBI-associated cognitive deficits and inflammatory mRNA expression in the cortex 7 dpi were independent of IFNAR1.

### Neuronal injury and cognitive deficits 7 dpi were mSTINGdependent.

Our data show that mSTING (and not IFNAR1) was important for inflammation after diffuse TBI. Moreover, our previous single cell and single nuclei RNA-seq studies show reduced homeostasis of cortical neurons 7 dpi was dependent on microglia [11, 22]. Based on these data, neuronal health/injury was assessed 7 dpi. First, NeuN^+^ signaling [37] and lipid debris (i.e., lipofuscin) in the cortex were assessed 7 dpi [26, 38]. For NeuN^+^ labeling in the cortex 7 dpi, percent area of NeuN^+^ was influenced by TBI and mSTING^−/−^ (Interaction, *F*_1,24_ = 9.68, *p*<0.005, Fig.6A&B). Post hoc analyses indicates that TBI-STING^+/+^ mice had the lowest NeuN^+^ expression compared to all groups including the TBI-mSTING^−/−^ mice (*p*<0.05, Fig.6B). Lipofuscin accumulation in the brain with age, disease, or brain injury may also represent reduced homeostasis of neurons and glia [38–40]. There was auto-fluorescent lipid debris visible in the cortex 7 dpi, especially within NeuN+ cells (Fig.6C). Quantification indicates that there tended to be increased auto-fluorescent lipid debris in cortical neurons (NeuN^+^) 7 dpi (*F*_1,19_ = 3.19, *p*=0.08, Fig.6D) that tended to be reduced by genotype (*F*_1,19_ = 3.93, *p*=0.06, Fig.6D). Thus, there was reduced NeuN^+^ signaling and increased lipofuscin in cortical neurons 7 dpi that was attenuated by mSTING^−/−^.

Next, neurofilament light chain (NF-L), a relevant biomarker of neural injury after TBI [40], was determined in the plasma of mice [29]. TBI increased NF-L protein (pg/ml) levels (*F*_1,23_ = 28.37, *p*<0.0001, Fig.6E), and this increase was influenced by mSTING^−/−^ (Interaction, *F*_1,23_ = 9.99, *p*<0.005). con rmed TBI-STING^+/+^ mice had the highest average NF-L expression (~3,000 pg/ml) in the plasma compared to all groups including the TBI-mSTING^−/−^ mice (*p*<0.005). We interpret these results to indicate that TBI increased neuronal damage and dysfunction in the cortex 7 dpi that was dependent on STING in microglia.

In a similar study, control and mSTING^−/−^ mice were subjected to control or TBI (mFPI), and cortical and hippocampal mediated cognition was assessed 7 dpi using NOR/NOL (Fig.6F–K). There were no differences in total time exploring the objects between groups (Fig.6F). Fig.6G–H shows TBI-induced reductions in exploration of the novel object 6 dpi (TBI, *F*_1,39_ = 24.53, *p*<0.0001). Time spent interacting with the novel object was influenced by TBI and mSTING^−/−^ (Interaction, *F*_1,39_ = 12.01, *p*<0.005, Fig.6G–H). Post-hoc analyses confirmed that TBI-STING^+/+^ mice spent the least amount of time with the novel object compared to all other groups, including the mSTING^−/−^ mice (*p*<0.0001, Fig.6G&H). These effects and interactions were mirrored in the NOL task at 7 dpi (Fig.6I–K). Time spent interacting with the novel object was influenced by TBI and genotype (Interaction, *F*_1,43_ = 10.81, *p*<0.005, Fig.6J&K). Post-hoc analyses confirmed that TBI-STING^+/+^ mice spent the least amount of time with the novel object compared to all other groups, including the TBI-mSTING^−/−^ mice (*p*<0.05, Fig.6J&K). Overall, microglial STING signaling was critical for neuronal dysfunction and cognitive impairment following TBI.

### Single nucleus RNA-sequencing of cortical neurons 7 days after TBI..

**Figure 7:**
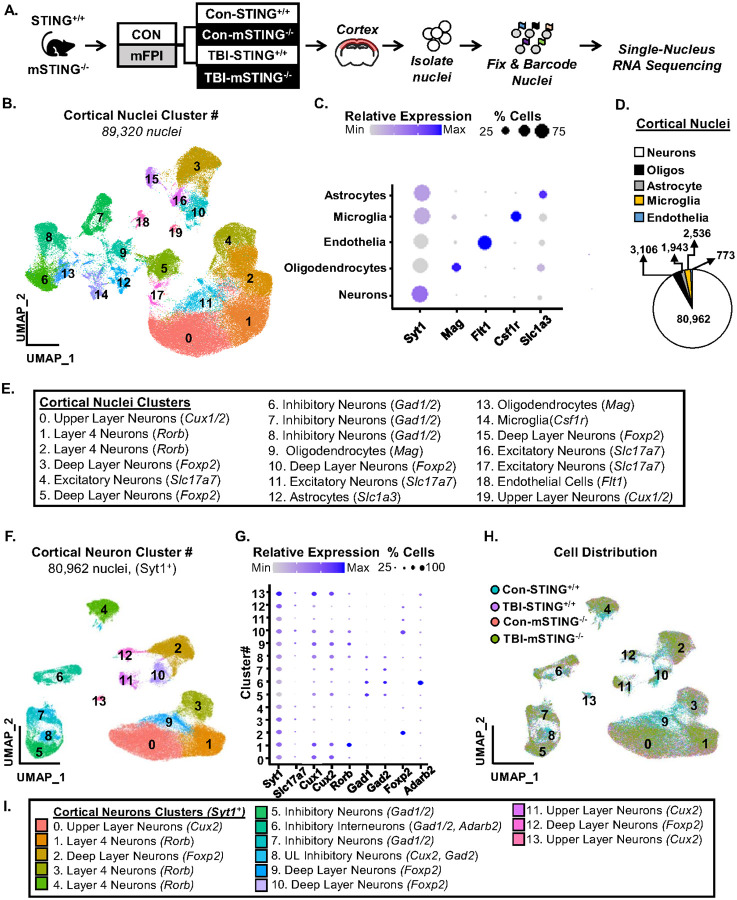
Single nucleus RNA-sequencing of cortical neurons 7 days after TBI. A) Male and female functional wild type (STING^+/+^) and CX_3_CR1/STING^−/−^ (mSTING^−/−^ mice were subjected to midline fluid percussion injury or left as uninjured controls. At 7 dpi, mice were sacrificed, and cortices were dissected, pooled (3 males, 3 females per group), and nuclei were collected. RNA profiles of collected nuclei were determined 7 dpi using snRNA-sequencing. Clustering and differential expression were determined using Seurat in R. B) UMAP plots indicate 19 distinct clusters of cortical cells based on identity genes. C&E) Clusters were identified based on established gene expression: neurons (*Syt1*), oligodendrocytes (*Mag*), microglia (*Csf1r*), astrocytes (*Slc1a3*) and endothelia (*Flt1*). Dot plot figure shows gene expression of identifying genes for clusters. D) Representative percentage of each cell type. Next, neurons (*Sty1+*) were subset and re-clustered. F) The UMAP plot shows that 14 clusters of cortical neurons identified. G&I) Dot plot figure shows gene expression of identifying genes for clusters based established gene expression: upper layer neurons (*Cux2*), layer 4 neurons (*Rorb*), deep layer neurons (*Foxp2*), and inhibitory neurons (*Gad1*). H) The UMAP plot shows the distribution of cortical neuron clusters between the experimental groups.

We have reported that microglia and type I interferon responses were associated with reduced neuronal homeostasis in the cortex 7 dpi [22]. Here, we aimed to determine the degree to which this was dependent on STING signaling from microglia. Thus, single nucleus RNA-sequencing (snRNA-seq) was conducted in cortical samples after control or TBI (7 dpi) in male and female STING^+/+^ or mSTING^−/−^ mice. Cortices were dissected, nuclei were isolated, fixed, and barcoded at 7 dpi (Fig.7A). Fig.7B shows that 89,320 nuclei were clustered into twenty distinct clusters. Clusters were identified based on gene expression of distinct markers (Fig.7C–E) (*Syt1*-neurons, *Slc1a3*-astrocytes, *Mag* – oligodendrocytes, *Flt1* – endothelia, and *Csf1r*–microglia). In line with previous work using snRNA-seq, 90% of cells detected in Fig.7D were neurons [22, 41, 42]. To delineate the neuronal profile with TBI and mSTING^−/−^ 7 dpi, cortical neurons were subset and re-clustered (Fig.7F) using existing gene markers (*Slc17a7, Cux1/2, Rorb, Gad1/2, Foxp2, Adarb2*) to classify the neuronal populations (Fig.7G&I). The distribution of cells based on the four experimental groups is shown (Fig.7H). Overall, there were approximately 80,000 nuclei collected from *Syt1*^+^ cortical neurons 7 dpi, and these nuclei were represented in all the experimental groups.

### Ablation of microglial STING attenuated the response to TBI in cortical neurons.

**Figure 8:**
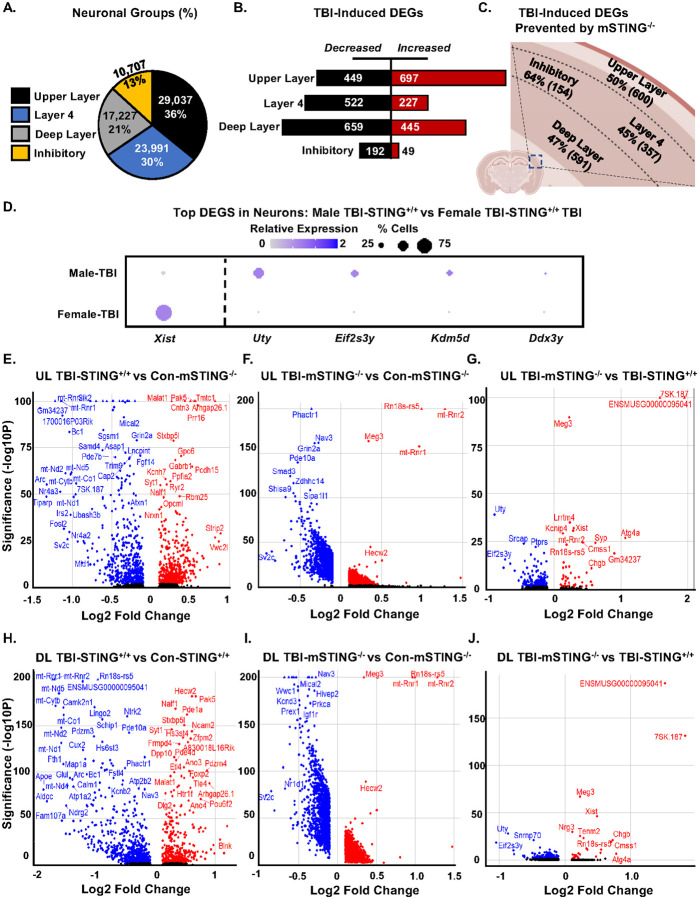
Ablation of microglial STING attenuated the response to TBI in cortical neurons. A) Continuing with the snRNA-seq experiment outlined in Fig.7, the pie chart shows the percentages of different Sty1+ neurons. B) The number of DEGs increased or decreased by TBI (TBI-STING^+/+^ vs Con-STING^+/+^) for each cortical neuron cluster are shown. C) The percentage and number of DEGs induced by TBI and prevented by mSTING−/− (TBI-STING^+/+^ vs TBI–mSTING^−/−^) in each neuronal cluster. D) Dot plot shows the top DEGs between male and female TBI-STING^+/+^ mice. E) Volcano plot of DEGs in the upper layer (UL) neurons of TBI-STING^+/+^ vs Con-TBI^+/+^. F) Volcano plot of DEGs in the upper layer (UL) neurons of TBI-mSTING^−/−^ vs Con-mSTING^−/−^. G) Volcano plot of DEGs in the upper layer (UL) neurons of TBI-mSTING^−/−^ vs TBI- STING^+/+^. H) Volcano plot of DEGs in the deep layer (DL) neurons of TBI-STING^+/+^ vs Con-TBI^+/+^. I) Volcano plot of DEGs in the deep layer (DL) neurons of TBI-mSTING^−/−^ vs Con-mSTING^−/−^. J) Volcano plot of DEGs in the deep layer (DL) neurons of TBI-mSTING^−/−-^vs TBI-STING^+/+^. Red dots represent genes significantly increased with |log2FoldChange| > 0 and *p*-adj <0.05. Blue dots represent genes significantly decreased with |log2FoldChange| > 0 and *p*-adj <0.05. Values were significant with a *p*-adj <0.05

Continuing with the snRNA-seq analyses of *Syt1*^+^ cortical neurons 7 dpi, the pie chart (Fig.8A) shows the distribution of specific neuronal profiles resolved. Consistent with our previous snRNA-seq experiments assessing cortical neurons [22], 36% of the *Syt1*^+^ nuclei corresponded to upper layer neurons (*Cux1/2*^+^), 30% of the *Syt1*^+^ nuclei corresponded to layer 4 neurons (*Rorb*^+^), 21% of the *Syt1*^+^ nuclei corresponded to deep layer neurons (*Foxp2*^+^), and 13% *Syt1*^+^ nuclei corresponded to inhibitory neurons (*Gad1/2*^+^). These neuronal sub-clusters were used for analyses. Fig.8B highlights that TBI resulted in both increased and decreased mRNA expression in cortical neurons 7 dpi, with more overall suppression of gene expression. For upper layer neurons, there were 1,146 DEGs (*p*-adj<0.05), with 697 increased and 449 decreased DEGs after TBI. Moreover, the influence of TBI on these upper layer neurons was 50% dependent on mSTING (600 DEGs, Fig.8C). For layer 4 neurons, there were 749 DEGs (*p*-adj<0.05) with 227 increased and 522 decreased DEGs after TBI. The influence of TBI on layer 4 neurons was 45% dependent on mSTING (357 DEGs, Fig.8C). For deep layer neurons, there were 1104 DEGs (*p*-adj<0.05) after TBI with 445 increased and 659 decreased DEGs. The influence of TBI on deep layer neurons was 47% dependent on mSTING (591 DEGs). Last, there were 241 DEGs (*p*-adj<0.05) in the inhibitory neurons with 49 increased and 192 decreased. In inhibitory neurons, the influence of TBI was 64% dependent on mSTING for 154 DEGs (*p*-adj<0.05, Fig.8C). Thus, there was a robust effect of TBI on cortical neurons 7 dpi and about 50% of the DEGs were prevented by mSTING^−/−^.

Notably, both male and female mice were included in these snRNA-seq studies. Although these studies were not appropriately powered to make comparisons based on sex, we examined male and female mice within the TBI-STING^+/+^ group. The top DEGs are shown in the dot plot for male TBI versus female TBI functional wild type mice (Fig.8C). *Xist* was increased in female wild type TBI mice compared to males and this is an X linked gene [43]. *Uty, Eif2s3y, Kdm5d*, and *Dxd3y* were increased in male TBI mice compared to female TBI mice. These genes are all y-linked [33, 44, 45]. Thus, male and female snRNA-seq data were analyzed together.

To visualize the significant DEGs in Fig8B&C, volcano plots are shown (Fig8E–J). For upper layer neurons (UL), the volcano plot shows the comparison between Con-STING^+/+^ and TBI-STING^+/+^ mice with 697 DEGs increased and 449 DEGs decreased (*p*-adj<0.05, Fig.8E). For instance, there was a TBI associated reduction in two synaptic plasticity related genes,

*Arc and Homer1*. Fig.8F shows the comparison between Con-mSTING^−/−^ and TBI-mSTING^−/−^ mice. In this comparison, 1728 DEGs were increased by TBI and 1312 DEGs were decreased. Fig.8G shows comparison between TBI-mSTING^−/−^ and TBI-STING^+/+^ mice. Here, there were 44 DEGs increased and 148 DEGs decreased. These data highlight a reduced influence of TBI on upper layer cortical neurons in the mSTING^−/−^ mice compared to controls (STING^+/+^ mice). For instance, the reduction of *Homer1* and *Arc* after TBI were prevented in the TBI-mSTING^−/−^ group.

For deep layer neurons (DL), the volcano plot shows comparison between Con-STING^+/+^ and TBI-STING^+/+^ mice with 445 DEGs increased and 659 DEGs decreased (*p*-adj<0.05, Fig.8H). For instance, there was a TBI associated reduction in three synaptic plasticity related genes, *Arc, Bdnf*, and *Homer1*. Reductions also evident in *ApoE* (lipid transport), *Calm1* (calcium signaling), and *Atg4a* (autophagy). Fig.8I shows the comparison between Con-mSTING^−/−^ and TBI-mSTING^−/−^ mice. There were 924 DEGs increased and 1662 DEGs decreased. These data highlight that there was a reduced influence of TBI on deep layer cortical neurons in the mSTING^−/−^ mice compared to controls (STING^+/+^ mice). For instance, the reduction of *Atg4a* and *Homer1* after TBI were prevented in the TBI-mSTING^−/−^ group (Fig.8J). Overall, TBI influenced the RNA profile of cortical neurons with a suppressive effect that was influenced by mSTING.

### Ablation of microglial STING attenuated TBI-induced imbalance in neuronal homeostasis of cortical neurons.

**Figure 9:**
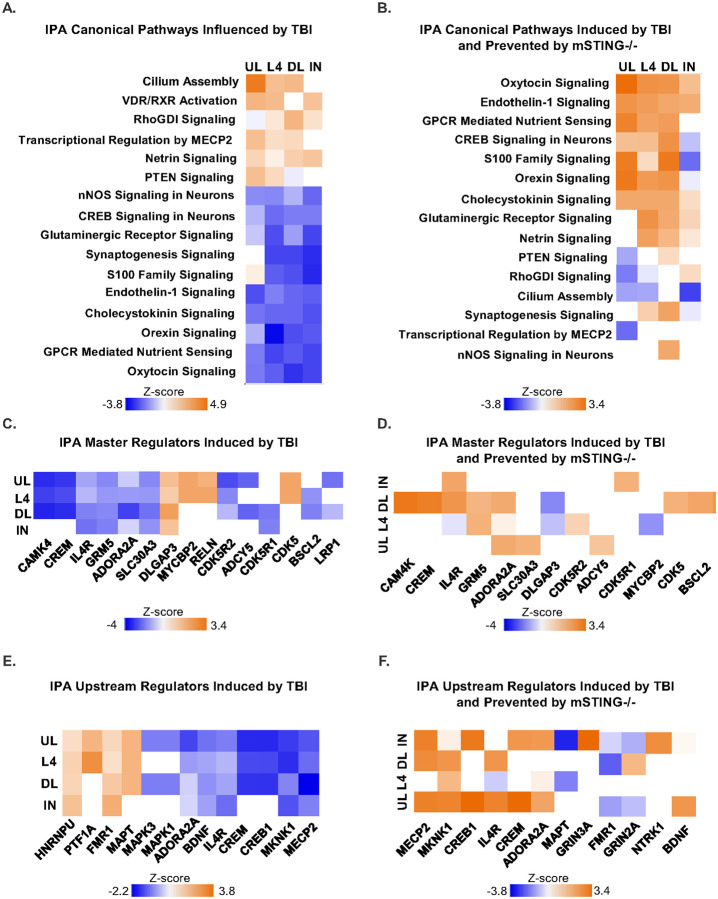
Ablation of microglial STING attenuated TBI-induced imbalance in neuronal homeostasis of cortical neurons. Continuing with the snRNA-seq experiment outlined in Figs.7&8. Ingenuity Pathway Analysis (IPA) assessed canonical pathways, master regulators, and upstream regulators influenced by TBI and mSTING^−/−^ in upper layer neurons (UL), layer 4 neurons (L4), deep layer neurons (DL), and inhibitory neurons (IN). A) Heat map of IPA canonical pathways induced by TBI (TBI vs Con-STING^+/+^) are shown. B) Heat map of IPA canonical pathways induced by TBI and prevented by mSTING^−/−^ are shown. C) Heat maps of activated or inhibited IPA master regulators by TBI (TBI vs Con-STING^+/+^). D) Heat map of activated or inhibited IPA master regulators by TBI and prevented by mSTING^−/−^ are shown. E) Heat map of activated or inhibited IPA upstream regulators induced by TBI are shown. Values were significant with a *p*-adj <0.05.

Continuing with the snRNA-seq analyses of *Syt1*^+^ cortical neurons 7 dpi, DEGs (*p*-adj<0.05) were analyzed in IPA for canonical pathways, master regulators and upstream regulators. Significant canonical pathways (z score, −3.8 to 3.9) influenced by TBI are shown in upper layer (UL), layer 4 (L4), deep layer (DL) and inhibitory (IN) cortical neurons (Fig.9A). These pathways were conserved across the four neuronal subtypes. For example, TBI increased canonical pathways associated with neuronal restructuring (e.g., Cilium Assembly, VDR/RXR Activation, RHOGDI Signaling, Transcriptional Regulation by MECP2, and Netrin Signaling) and inhibition of growth (PTEN signaling). TBI also suppressed canonical pathways associated with neuronal homeostasis and metabolism (CREB Signaling, Synaptogenesis, S100 Family Signaling). These data are consistent with our previous work on the effects of TBI on neurons [22]. Next the significant canonical pathways induced by TBI and prevented by mSTING are shown in upper layer (UL), layer 4 (L4), deep layer (DL) and inhibitory (IN) cortical neurons (Fig.9B). Canonical pathways that were decreased following TBI and influenced by mSTING were associated with neuronal health (Oxytocin Signaling, Endothelin-1 Signaling, CREB Signaling, and Orexin Signaling). Canonical pathways that were increased by TBI associated with neuronal restructuring (Cilium Assembly) and inhibition of growth (PTEN) were also prevented by mSTING^−/−^ (Fig.9B).

Next, significant master regulators that were influenced by TBI are shown in upper layer (UL), layer 4 (L4), deep layer (DL) and inhibitory (IN) cortical neurons (Fig.9C). TBI increased DLGAP3, MYCBP2, RELN, CDK5 in upper layer and layer 4 neurons, and decreased CAMK, CREM, IL-4R, and GRM5. Master regulators reduced by TBI associated with neuronal homeostasis (e.g., CAM4K, CREM, IL4R, GRM5, ADORA2A) were prevented by mSTING, especially in deep layer neurons (Fig.9D). As such, deep layer neurons had the most master regulators prevented by mSTING^−/−^ (7). Upper layer neurons had the most upstream regulators induced by TBI and prevented by mSTING^−/−^ (7). These upstream regulators are associated with neuronal homeostasis (e.g., MECP2, MKNK1, CREB1, IL4R, CREM, ADORA2A, BDNF). These regulators were reduced by TBI, and this reduction was prevented by mSTING^−/−^ (Fig.9F). Upstream regulators increased by TBI include HNRNPU, PTF1A, FMR1, and MAPT and decreased upstream regulators include BDNF, IL4R, CREM, and CREB1 (Fig.9E). These changes were prevented by mSTING, especially in upper layer cortical neurons (Fig.9F). Taken together, TBI reduced cortical neuronal homeostasis, and this was dependent on STING in microglia 7 dpi.

## Discussion

We previously reported that a global knockout of the stimulator of interferons genes (STING) reduced chronic inflammation and cognitive impairment associated with diffuse TBI [22]. Thus, the aim of this study was to determine the degree to which a microglia-specific knockout of STING influenced neuroinflammation, neuronal dysfunction, and cognitive deficits induced by diffuse TBI. Here, TBI induced microglial morphological restructuring and cortical inflammation 7 dpi were mSTING dependent. In addition, neuronal injury and cognitive impairment 7 dpi were also dependent on mSTING. With snRNA-seq of cortical neurons after TBI, there were reductions in CREB signaling, synaptogenesis, and oxytocin signaling and increases in cilium assembly and PTEN signaling. These reductions in neuronal homeostasis were mSTING dependent. Collectively, ablation of STING in microglia attenuated TBI-induced IFN-dependent responses, cortical inflammation, cortical pathology, neuronal dysfunction, and cognitive impairment.

One key finding of this study was that increased STING expression 7 days after TBI in the cortex was localized to IBA1^+^ microglia, and this increase in STING was ablated by microglial STING^−/−^. The increase in STING expression in the cortex after TBI is consistent with previous findings showing enhanced IFN-I responses after either diffuse [11, 15, 16, 22, 36] or penetrating TBI [23, 24, 35, 46]. Moreover, studies of penetrating TBI induced by controlled cortical impact (CCI) indicate that STING is localized to IBA1^+^ microglia [23, 24, 46]. The extension here is that STING was localized to cortical microglia after diffuse TBI and this increase was ablated by a transgenic model of mSTING^−/−^. While STING induction was detected in other cell types including neurons and astrocytes after TBI [23, 24, 46, 47], STING after diffuse TBI was localized in cortical microglia and undetectable in astrocytes and neurons. Assessment of mRNA from percoll enriched microglia paralleled these data with a TBI-dependent increase in STING mRNA 7 dpi and ablation by microglial STING^−/−^. These findings are also consistent with our previous reports using snRNA-seq that microglia, not neurons, expressed genes associated with the production of IFN-I after TBI [22]. These RNA and protein findings validate the targeted knock out of STING in microglia. Overall, TBI increased STING expression within IBA1^+^ microglia 7 dpi was ablated in mSTING^−/−^ mice.

Another relevant point is that TBI-induced microglial restructuring (IBA1^+^ percent area increase) 7 dpi was dependent on mSTING. Rod-shaped microglia and GFAP^+^ astrocytes were also increased 7 dpi, but were independent of mSTING^−/−^. These data are similar to our previous reports where TBI-induced microglial restructuring was reduced by global STING^−/−^, but astrogliosis was unaffected [22]. In addition, another report showed that astrocytes were unresponsive to STING activation after TBI [16]. Rod-shaped microglia are detected in humans and rodents in the context of advanced age, neurodegeneration, and TBI [12, 31, 48], but their function is unclear. In a previous report, rod-shaped microglia were reduced in the cortex 7 dpi of global STING^−/−^ mice [22]. Here, rod-shaped microglia were unaffected by mSTING^−/−^. One explanation for this difference is that rod-shaped microglia in the cortex 7 dpi are mSTING independent and may serve a neuroprotective role. For instance, these structurally unique and elongated microglia aligned with apical dendrites of damaged neurons in the cortex 7 dpi [12]. These cells were present 7 dpi in the cortex of mice with microglia depletion (PLX5622), which was associated with reduced neuroinflammation and cognitive improvement [11]. Furthermore, elimination of rod-shaped microglia using a TREM2 knock out in an ALS model increased neuronal hyperactivity, worsened motor deficits, and further reduced survival rates in mice [49]. Collectively, there were structurally divergent pro les of cortical glia 7 dpi and the reactive microglia pro le was attenuated by mSTING^−/−^.

Another point for discussion is the increased IFN-I and pro-inflammatory signaling in the cortex 7 dpi was dependent on microglial STING. For example, there were 232 genes detected in the NanoString panel (770 genes) associated with type I interferon signaling, inflammation, and antigen presentation 7 dpi. A majority of these TBI-associated genes were reduced (86%) by mSTING^−/−^. Notably, there were minimal sex differences detected in the cortical mRNA analyses with only two sex-linked genes (*Kdm5d* and *Uty*) [33, 44] different between male and female TBI mice. Overall, TBI increased genes associated with IFN-I and inflammation in male and female mice, and these were reduced by mSTING^−/−^. Consistent with these DEGs, IPA canonical pathways and master regulator analyses showed myriad IFN-I and inflammatory pathways increased after TBI including activation of IRF, NFκB, and cGAS-STING. These increases in genes and pathways associated with IFN-I, inflammation, and microglial priming are consistent with previous reports 7 dpi [12, 16, 22]. Key pathways induced by TBI 7 dpi and prevented by mSTING^−/−^ included cGAS-STING, NF-κB, and neuroinflammation signaling. Notably, some DEGs (35) and IPA pathways that were induced by TBI were unaffected by mSTING^−/−^. These DEGS were genes associated with the complement cascade (*C3, C4a*), astrocyte associated genes (*Aldh1l1, Gja1*) and endothelia associated genes (*Blnk, Enpp6*). There were 12 total genes uniquely increased by TBI in mSTING^−/−^ mice. A majority of these increased DEGs were neuronal (*Tubb3, Gria, Slc17a7, Rala, etc*.), and may represent improved neuroprotection following TBI in mSTING^−/−^ mice. Thus, the inflammatory and IFN-I responses in the cortex 7 dpi were robustly influenced by STING in microglia.

One notable finding of this study was that global IFNAR1 knockout did not reduce cortical inflammation or cognitive impairment 7 dpi. We and others have shown increased genes and pathways following diffuse and penetrating TBI related to the IFNAR1 pathway [11, 15–17, 22–24, 35, 36]. Presumably cGAS-STING activation in microglia after TBI increases *Irf3* and corresponding IFN-I that would use the IFNAR1 [19, 50]. Indeed, several studies show improvements in inflammation, cognition, and neurologic dysfunction following selective modulation of the IFNAR1 pathway with diffuse [36] and penetrating TBI [35]. Here, global IFNAR1 knockout did not reduce cortical in ammation or cognitive impairment 7 dpi. Global IFNAR1 knockout, however, reduced the induction of *Irf7* 7 dpi. One explanation is that the STING pathway also promotes NF-κB-mediated genes (*e.g., IL-6, TNF* and *IL-1*) [20, 21] and these pro-inflammatory cytokines are more responsible for the downstream effects on neurons and cognitive processes. For instance, the IL-1 receptor-1 (IL1-R1) is highly expressed on DG neurons of the hippocampus [41, 51] and IL-1/IL-R1 responses are evident chronically after closed head TBI [52]. Another explanation is that there are reported confounds of IFNAR1^−/−^. For instance, one report showed that global and microglia specific knockouts of IFNAR1 led to dysfunctional microglia with a “bubble” phagosome formation and increased accumulation of DNA-damaged neurons [53]. Another study showed that astrocytic IFNAR1 deletion in mice caused cognitive dysfunction and reduced synaptic plasticity [54]. Because of the potential confounds of these global IFNAR1^−/−^ mice, we conducted only limited studies with them and instead focused on mSTING^−/−^ mice. Taken together, the interpretation is that ablating STING in microglia was more beneficial than targeting IFNAR1 because STING is upstream and thus affects both IFN-I and NF-κB mediated responses after diffuse TBI.

Another relevant finding was the neuropathological influences of TBI (7 dpi) were dependent on STING in microglia. For example, there was reduced percent area labeling of NeuN^+^ in the cortex 7 dpi, which was attenuated in TBI-mSTING mice. The interpretation is that reduced NeuN^+^ labeling corresponds with more dysfunction or atypical neurons in the cortex after diffuse TBI. A similar TBI-induced reduction of NeuN in the cortex 7 dpi was detected in a weight drop model of TBI in mice (up to 6 months later) and associated with increase blood brain barrier permeability after TBI [37]. In the same study, the reduced NeuN^+^ neurons were associated with reduced synaptic plasticity [37]. Parallel to this, lipofuscin (i.e., autofluorescent lipid debris) detected here in cortical neurons may also represent reduced homeostasis [55]. Indeed, several studies show increased lipofuscin in the brain with age or after TBI [26, 38]. Moreover, increased lipofuscin after TBI in aged mice was associated with neuronal loss, glial activation, and oxidative stress [38]. Taken together, targeted mSTING deletion prevented in ammatory cytokine, chemokine, and IFN-I production that deleteriously affected neuronal homeostasis in the cortex.

Consistent with the above data, we show novel data that the TBI-associated increase in plasma NF-L (7 dpi) was attenuated in the TBI-mSTING^−/−^ mice. NF-L is a clinically validated biomarker for neuronal and axonal injury after moderate to severe TBI in humans [40, 56]. Moreover, use of plasma NF-L as a biomarker reflecting the extent of underlying neuropathology in humans has been validating using MRI and cerebral microdialysis [40]. Thus, we interpret the data to show ablating STING in microglia was neuroprotective with less axonal and neuronal damage after diffuse TBI. Non-selectively inhibition of microglia after CNS injury may have off target effects that worsen recovery. For instance, minocycline reduced microglia activation in humans (by MRI) after TBI, but increased the neuronal damage marker, NF-L, in the plasma [57]. Furthermore, depletion of microglia prior to spinal cord injury worsened pathology by interfering with astrocytic dynamics [58]. Thus, inhibition of specific microglia pathways, like STING, are critical for addressing chronic neuroinflammation elicited by traumatic CNS injury while minimizing off target effects of treatment. Parallel with the evidence of increased neuronal injury 7 dpi, TBI reduced cortical/hippocampal dependent memory with reduced novel object/exploration 7 dpi. Here, novel data shows that these reductions in cognition after TBI were mSTING dependent. These data are consistent with previous studies of global STING^−/−^ [22] and microglial elimination [11] showing that limiting inflammatory pathways in microglia improved behavioral and cognitive recovery after diffuse TBI. Taken together, TBI induced neuronal and cognitive dysfunction 7 dpi associated with increased NF-L, reduced NeuN^+^ expression, and cognitive de cits were prevented by mSTING^−/−^.

Consistent with our previous data [11, 22] snRNA-seq analysis in the cortex 7 dpi shows suppression of neuronal pathways associated with metabolism and homeostasis (CREB Signaling in Neurons, Synaptogenesis, S100 Signaling, GPCR Mediated Nutrient Sensing, and Cholecystokinin Signaling). This pattern was conserved across all neuronal subtypes sampled, especially the excitatory neurons (DL, L4, and UL) indicating a shared pattern of cortical neuron suppression. Here, novel data shows that t extension was that STING ablation in microglia prevented these imbalances. For instance, approximately 50% of all DEGs influenced by TBI were prevented in microglial mSTING^−/−^ mice. The mSTING dependent reversals of the TBI effects in upper layer (UL), layer 4 (L4), and deep layer (DL) neurons included canonical pathways (Cilium Assembly, RHODI, and Netrin Signaling) and master regulators (HNRNPU, PTF1A, FMR1, MAPT) associated with neuronal restructuring. These RNA data are consistent with the physiological neuronal restructuring and dendritic atrophy detected after TBI [11]. These physiological changes reported after TBI in mice were associated with cognitive dysfunction and depressive-like behavior [10, 11]. In addition, the mSTING dependent reversals of the TBI effects in UL, L4, and DL neurons included canonical pathways (Oxytocin Signaling, GPCR Mediated Nutrient Sensing, CREB Signaling, S100 Family Signaling) and master regulators (MECP2, CREB1, IL4R, and BDNF) associated with neuronal homeostasis and metabolism. These pathways and master regulators increased following TBI are related to neuronal and synaptic remodeling, and likely represent the same cassette of genes previously reported to be associated with the Phosphatase and Tensin Homolog (PTEN) signaling [22]. PTEN is a master regulator of neuronal and dendritic morphological restructuring [59], and the increase in PTEN following traumatic CNS injuries is a potential target of intervention [60, 61]. In summary, the reduced homeostasis of neurons in the cortex 7 dpi was associated with increased STING responses in microglia.

A final point for discussion is that this study was not powered for sex comparisons. Nonetheless, male and female mice were used and there were similar patterns of responses following TBI. For instance, NanoString and single nucleus RNA-seq analyses revealed the same sex-linked genes (*Uty, Kdm5d*) were the top DEGs between male and female mice 7 dpi. Again, sex differences in TBI is an important issue and several reports indicate sexually dimorphic responses following TBI [62–65]. Nonetheless, STING and IFN-I responses to diffuse TBI were conserved in male and females 7 dpi. In conclusion, while sex differences are important aspects to interrogate with TBI, STING, and IFN-I responses after diffuse TBI were conserved in males and females.

In summary, we show that diffuse TBI induced a STING response in microglia associated with IFN-I that impairs cortical neuronal homeostasis and cognition. The TBI-induced neuronal restructuring, neuronal damage, and snRNA-profiles were dependent on microglial STING. Targeted pharmacotherapies to reduce this microglial STING response may be beneficial in reducing neuroinflammation and corresponding neurocognitive complications following TBI.

## Data Availability

The data that support the findings of this study are available from the corresponding author upon reasonable request.
